# Starch Production in *Chlamydomonas reinhardtii* through Supraoptimal Temperature in a Pilot-Scale Photobioreactor

**DOI:** 10.3390/cells10051084

**Published:** 2021-05-01

**Authors:** Ivan N. Ivanov, Vilém Zachleder, Milada Vítová, Maria J. Barbosa, Kateřina Bišová

**Affiliations:** 1 Laboratory of Cell Cycles of Algae, Centre Algatech, Institute of Microbiology of the Czech Academy of Sciences, 379 81 Třeboň, Czech Republic; ivanov@alga.cz (I.N.I.); zachleder@alga.cz (V.Z.); vitova@alga.cz (M.V.); 2 Faculty of Science, University of South Bohemia, Branišovská 1760, 370 05 České Budějovice, Czech Republic; 3 Bioprocess Engineering Group, AlgaePARC, Wageningen University & Research, P.O. Box 16, 6700 AA Wageningen, The Netherlands; maria.barbosa@wur.nl

**Keywords:** microalgae, *Chlamydomonas reinhardtii*, starch, supraoptimal temperature, cell cycle, pilot-scale production

## Abstract

An increase in temperature can have a profound effect on the cell cycle and cell division in green algae, whereas growth and the synthesis of energy storage compounds are less influenced. In *Chlamydomonas reinhardtii,* laboratory experiments have shown that exposure to a supraoptimal temperature (39 °C) causes a complete block of nuclear and cellular division accompanied by an increased accumulation of starch. In this work we explore the potential of supraoptimal temperature as a method to promote starch production in *C. reinhardtii* in a pilot-scale photobioreactor. The method was successfully applied and resulted in an almost 3-fold increase in the starch content of *C. reinhardtii* dry matter. Moreover, a maximum starch content at the supraoptimal temperature was reached within 1–2 days, compared with 5 days for the control culture at the optimal temperature (30 °C). Therefore, supraoptimal temperature treatment promotes rapid starch accumulation and suggests a viable alternative to other starch-inducing methods, such as nutrient depletion. Nevertheless, technical challenges, such as bioreactor design and light availability within the culture, still need to be dealt with.

## 1. Introduction

Together with light and nutrient availability, temperature is one of the major abiotic factors affecting growth of microalgae [1,2,3,4]. Temperature has been found to affect individual metabolic processes in various ways. Cell division and the duration of the cell cycle are particularly susceptible to changes in temperature while other parts of cellular metabolism, such as growth and other related synthetic processes, appear to be less influenced by such changes [5,6].

In green algae dividing by multiple fission, a gradual increase in temperature results in a physiological response in which cells at first increase their growth rate and shorten their cell cycle. Upon a further increase in temperature, an optimal point is reached at which growth rate is at its maximum and cell cycle duration is at its minimum. However, after exceeding this optimal temperature, the duration of the cell cycle is gradually prolonged while growth rates remain unaffected [1]. Eventually, after reaching a certain temperature (hereafter referred to as supraoptimal), the cell cycle is completely blocked while growth and metabolism remain seemingly further unaffected [1,6]. This effect of supraoptimal temperature was first observed in the 1960s and 1970s during small-scale laboratory experiments conducted with *Chlorella* sp. (Chlorophyta) [6,7,8,9]. It was determined that the specific supraoptimal temperature that causes cell cycle arrest varies between species of microalgae and must be controlled within a very narrow range. Otherwise, the cells will not achieve cell cycle arrest (at a temperature lower than supraoptimal) or will have their metabolism strongly affected which might lead to cell death (at a temperature higher than supraoptimal) [6].

An inherent property of cell division is that it is an energy-demanding process, consuming the majority of the cell’s energy reserves [10]. A simple block of cell division leads to accumulation of starch and/or lipids in microalgal cultures grown in nitrogen (and other nutrient) starvation or limiting conditions [11,12,13,14,15]. A combination of cell cycle arrest and unaltered growth metabolism, as is the case of supraoptimal temperature treatment, leads to the build-up of surplus energy reserves [6,9]. For starch producing green algae, the accumulation of starch under supraoptimal temperature can be extensive and it can reach levels considerably higher than in cells cultivated at the optimal growth temperature and hence, it can be utilized as an approach to increase starch productivity.

*Chlamydomonas reinhardtii* has served as a well-established model for a number of years [13,16]. This green alga benefits from a wide array of readily available molecular tools for genetic engineering and strain optimization [16,17,18]. However, in spite of these benefits, the adoption of *C. reinhardtii* as a biotechnology platform has been limited. Only recently, attempts were made to increase the starch content of *C. reinhardtii* by utilizing techniques such as nutrient deprivation and temperature stress [6,10,13,19,20,21,22]. Although nutrient deprivation is an effective technique that can increase the starch content of *C. reinhardtii* to almost 49% (*w*/*w*), the build-up is rather slow and it can take weeks until the maximum concentration is reached, which reduces overall productivity [22]. In contrast, temperature stress can provide a rapid method for starch accumulation within short periods of time. In laboratory-scale experiments with synchronized cultures of *C. reinhardtii,* the cells at 30 °C initially accumulated starch as they grew in size, but this starch was spent for cell division. The cells at 39 °C grew in size similar to those at 30 °C, but they did not divide. Instead, they continued to increase their cell size and after 24 h, their total starch content was more than two-fold higher than the maximum at 30 °C [6]. Although promising, these results were obtained only under controlled laboratory conditions that utilized synchronized cultures with relatively low biomass densities and were exposed to abundant light intensities. Hence, the applicability of the supraoptimal temperature method for industrial production of starch is still largely unknown.

In the present study, we examine the potential for pilot-scale starch production in *C. reinhardtii* by supraoptimal temperature, a method that has already been proven to cause a rapid 2-fold increase in starch yields under laboratory conditions [6]. In doing so we investigate whether and how biomass density affects starch productivity, the possibility of culture recovery and reuse after the supraoptimal temperature treatment, as well as potential practical challenges and limitations of the method. To our knowledge, the experiments described here are the first attempt to employ supraoptimal temperature in the production of starch in microalgae at pilot-scale.

## 2. Materials and Methods

### 2.1. Microorganism and Culturing Conditions

The algal strain used in these experiments was the unicellular alga *Chlamydomonas reinhardtii* wild type 21gr (CC-1690), obtained from the Chlamydomonas Resource Center at the University of Minnesota (St. Paul, MN, USA). For routine subculturing, the strains were streaked onto culture plates containing standard high salt (HS) medium [23] solidified with agar every three weeks.

For the purpose of the experiments, a starting culture was cultivated in a bench-top flat-panel airlift photobioreactor (Algaemist, Technical Development Studio, Wageningen University, Wageningen, The Netherlands) in the following manner: 400 mL of liquid HS medium was inoculated directly from the culture plates and was cultivated at 30 °C and under constant incident light intensity of 500 µmol photons m^−2^ s^−1^ of photosynthetically active radiation (PAR) provided by light-emitting diode lamps (BXRA W1200, Bridgelux, Fremont, CA, USA). The cultures were aerated with a mixture of air and CO_2_ (2%, *v*/*v*) at a flow rate of 0.63 VVM in order to provide a carbon source and mixing of the cell suspension.

### 2.2. Culture Medium for the Pilot-Scale Cultivation

All experiments were performed under photoautotrophic conditions. The culture medium used for the pilot-scale experiments was based on a HS medium but was modified in order to facilitate high biomass yields with the NH_4_Cl concentration being increased 5-fold. This resulted in a growth medium with the following final composition: 250 g L^−1^ NH_4_Cl, 2 CaCl_2_·2H_2_O, 20 g L^−1^ MgSO_4_·7H_2_O, 1.84 g L^−1^ C_10_H_12_FeN_2_NaO_8_, 0.05 g L^−1^ Na_2_MoO_4_·2H_2_O, 3.09 g L^−1^ H_3_BO_3_, 1.18 g L^−1^ MnSO_4_·7H_2_O, 1.40 g L^−1^ CoSO_4_·7H_2_O, 1.24 g L^−1^ CuSO_4_·5H_2_O, 1.43 g L^−1^ ZnSO_4_·7H_2_O, 72 g L^−1^ KH_2_PO_4_, 134 g L^−1^ K_2_HPO_4_. For the preparation of the medium, 100× concentrated stock solutions of macro elements and microelements were used. All components, excluding phosphates, were diluted in distilled H_2_O and autoclaved for 20 min at 121 °C. After cooling, the sterile autoclaved phosphates were added. The medium used for cultivation in the pilot-scale flat-panel photobioreactor was not sterilized and tap water was used to dilute the stock solutions. In the course of the experiments, pH was monitored daily and was maintained at 7.0 ± 0.1 by the addition of 2 M NaOH. Foam formation in the reactor vessels was controlled with the help of 10× diluted antifoam silicone Snapsil RE 20 containing 30% active compound (Product code: 84538.290, VWR International, LLC, Radnor, PA, USA).

### 2.3. Pilot-Scale Flat-Panel Photobioreactor

**Figure 1:**
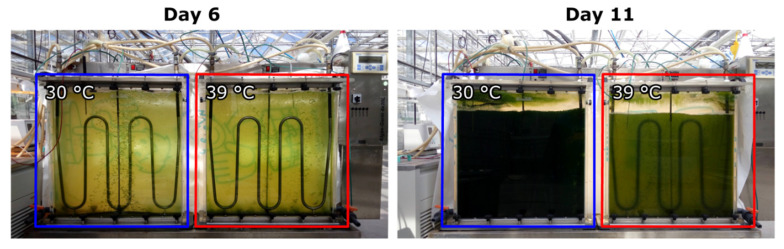
Changes of optical densities of *C. reinhardtii* cultures starting at the same cell density (day 6, left photobioreactor) and grown at the same light intensity for 5 days at temperatures of 30 °C and 39 °C (day 11, right photobioreactor). For description of the photobioreactor see Materials and Methods in Section 2.3.

A flat-panel Algae-Germ photobioreactor with two cultivation vessels, each of a total volume of 25 L (20 L of culture volume) (Figure 1), was used in all experiments described here. The photobioreactor was situated at 51°59′45.6″ N, 5°39′25.7″ E in Wageningen, Netherlands and was placed within a greenhouse with panels facing 240° SW. Each of the cultivation vessels had the following dimensions: length: 70 cm, height: 72 cm, width (optical path: 5 cm). Cooling and heating of the microalgal suspension culture was provided by two refrigerating/heating circulators (Julabo GmbH, Seelbach, Germany) that circulated water through temperature control coils, which were submerged in the culture suspension. A simple aeration system provided a constant flow of a mixture of air and CO_2_ (2%, *v*/*v*) and ensured mixing of the suspension culture. Both cultivation vessels were constantly illuminated by a panel of luminescent lamps (Master TL-D 58W/840, Philips, Amsterdam, the Netherlands) delivering 50 µmol photons m^−2^ s^−1^ of PAR (measured at the vessel surface). However, the majority of PAR delivered to the cultures was through exposure to natural sunlight.

### 2.4. Experimental Approach

All the experiments were performed in the time-span between July 17th and September 17th, 2018 (Table 1). Each experiment consisted of two phases. During the biomass accumulation phase, one cultivation vessel was filled with 20 L of HS medium and was inoculated with 0.8 L of starting inoculum of cell concentration approximately 3.5 × 10^7^ cells mL^−1^. The resulting culture, with an initial cell concentration of approximately 1.4 × 10^6^ cells mL^−1^, was then incubated at 30 °C for 4 to 6 days. The supraoptimal temperature phase started upon reaching a biomass concentration exceeding 1.0 g L^−1^. At this point the culture was diluted with HS medium and was separated into two cultivation vessels, which were then transferred to 30 °C or 39 °C.

### 2.5. Light Measurements

The photon flux density (Iph, µmol photons m^−2^ s ^−1^) was measured with a LI-COR 190-SA 2π PAR (400–700 nm) quantum sensor (LiCor, Lincoln, NE, USA). Continuous light data logging was made with a sensor from the same model and manufacturer, mounted outdoors and facing the sky, parallel to the ground.

To obtain a measure of light energy absorbed by the cell suspension grown at different concentrations of cells, the mean light intensity (I) was calculated according to the Lambert-Beer law:

I = (I_i_ − I_t_)/ln(I_i_/I_t_),

where I_i_ is the incident light intensity at the surface of the culture vessel and I_t_ is the transmitted light intensity measured at the rear side of the culture vessel. The mean light (µmol cell^−1^) was calculated by dividing the mean light intensity during 24 h (obtained by continuous light data logging) by the number of viable cells for that period. 

### 2.6. Cell Size and Cell Number Measurements

One milliliter aliquots of culture suspension were taken, fixed with 10 µL of iodine solution (1 g I, 5 g KI, 100 mL H_2_O), and stored at 4 °C. Cell diameter was measured on microphotographs taken with an Olympus Camedia C-5050 Zoom digital camera. The microphotographs were then analyzed using ImageJ image processing and analysis software (U. S. National Institute of Health, Rockville Pike, MD, USA). The cell diameter was recalculated to volume by a basic formula assuming a spherical cell shape. Cell number was determined by means of a Bürker counting chamber (Meopta, Prerov, Czech Republic).

### 2.7. Dry Matter Measurements

Aliquots of culture suspension (50 mL) were taken and centrifuged (1580R, Labogene ApS, Lillerød, Denmark) for 10 min at 4000 rpm. The supernatant was then removed, and the remaining pellet was transferred to a 2 mL preweighed test tube and dried for 24 h at 105 °C. After cooling for 2 h, the test tube with the pellet was weighed on an analytical balance (CP224S-OCE, Sartorius AG, Göttingen, Germany) and the weight of the pellet was determined by subtracting the weight of the empty test tube.

### 2.8. Starch Analysis

A starch-specific enzymatic method was used to accurately estimate the biomass starch content. Aliquots of culture suspension (10 mL) were harvested and centrifuged (1580R, Labogene ApS, Lillerød, Denmark) for 10 min at 4000 rpm. The supernatant was then discarded, and the resulting pellets were stored at −20 °C. After thawing, the cells in the pellets were disintegrated by adding 300 µL of glass beads (0.7 mm in diameter) and vortexing vigorously (MS3, IKA‑Werke GmbH & Co. KG, Staufen, Germany) for 15 min. Depigmentation of the samples was carried out by adding 1 mL of 80% (*v*/*v*) ethanol to the pellet and incubating in a water bath for 15 min at 68 °C after which the samples were centrifuged (1580R, Labogene ApS, Lillerød, Denmark) for 2 min at 14,000 rpm and the supernatant was removed. The depigmentation procedure was repeated 3 to 4 times (or until the pellet was completely discolored). After that, 1 mL of α‑amylase (porcine pancreas, Sigma–Aldrich, St. Louis, MO, USA) solution (0.5 g·L^−1^ *w*/*v* in 0.1 M sodium phosphate buffer (pH 6.9)) was added to the samples and incubated for 1 h at 37 °C. The samples were centrifuged (1580R, Labogene ApS, Lillerød, Denmark) for 2 min at 14,000 rpm, after which the supernatant was used for the quantification of reducing sugars through the dinitrosalicylic acid (DNSA) color reaction, as described by Miller [24]. Briefly, 500 µL of supernatant were mixed with 500 µL DNSA solution (1% (*w*/*v*) DNSA, 30% (*w*/*v*) potassium sodium tartrate tetrahydrate, 20% (*v*/*v*) 2 M sodium hydroxide) and incubated for 5 min at 105 °C on a heat block. Following a cooling period of 10 min at room temperature the mixture was diluted five-fold with distilled water, after which the absorbance of the samples was measured at 570 nm. The concentration of starch was estimated through a calibration curve of potato starch (Lach-Ner s.r.o., Neratovice, Czech Republic) digested with α-amylase.

### 2.9. Microscopic Observations and Starch Staining

Microscopic observations during the course of the experiments were carried out daily on a Leica Laborlux S microscope. Staining of starch was with the same iodine solution that was used for fixing cell counting samples in a 1:10 volume ratio of staining solution to sample.

## 3. Results

Starch accumulation in *C. reinhardtii* can be induced by nutrient depletion [12,13,22]. In order to exclude such effect, a biomass accumulation phase in fully supplemented medium at the optimal temperature was included prior to the supraoptimal temperature phase. The purpose of the biomass accumulation phase was to demonstrate that the cultures were not limited by nutrients and to estimate the typical starch content in *C. reinhardtii* cultures during exponential growth under the optimal growth temperature. Please, refer to Table 1 for an overview of the time span of the experiments as well as combinations of biomass densities and temperature treatments applied.

### 3.1. The Effects of Supraoptimal Temperature 

To assess the effects of supraoptimal temperature on cell growth and division, as well as starch accumulation, the culture behavior at two parallel treatments of 30 °C (control) and 39 °C was compared. At first a *C. reinhardtii* culture was cultivated at 30 °C for 6 days. After reaching a biomass concentration of 1.0 g L^−1^, the culture was diluted with HS medium to 0.1 g L^−1^ and split into two cultures which were then cultivated at 30 °C and 39 °C, respectively (Figure 1).

**Figure 2:**
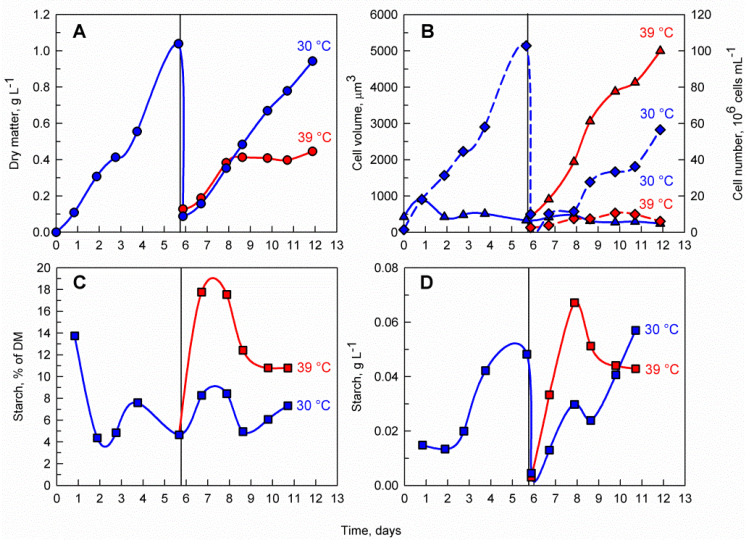
Effect of supraoptimal temperature on dry matter accumulation (**A**), average cell volume (solid line, triangles) and cell number (dashed line, diamonds) (**B**), biomass starch content (**C**) and volumetric starch concentration in the culture (**D**). The vertical line on day 6 represents the shift from biomass accumulation phase to supraoptimal temperature phase. Blue lines represent cultivation at 30 °C while red lines represent cultivation at 39 °C. During the biomass accumulation phase a single *C. reinhardtii* culture was cultivated at 30 °C. After 6 days the culture was diluted to a biomass concentration of 0.1 g L^−1^ and divided into two separated cultures. One of the cultures was cultivated at 39 °C (supraoptimal temperature treatment) while the other one was cultivated at 30 °C (control). Panel B represents the median volume of cells and an estimation of the mean cell number within a defined volume of culture suspension.

The control culture cultivated at 30 °C had a similar pattern of biomass accumulation to that before dilution and returned to the pre-dilution biomass concentration within 6 days (reaching a maximum of nearly 1 g L^−1^) (Figure 2A). In contrast, biomass accumulation of the culture at 39 °C stopped after 2 days, reaching a maximum of only 0.4 g L^−1^. The biomass starch content in terms of percentage of starch within the dry matter (DM) was much greater in the culture cultivated at 39 °C, reaching 18% of DM as opposed to 8% of DM at 30 °C (Figure 2C). Moreover, the volumetric starch concentration in mg mL^−1^ was faster in the culture cultivated at 39 °C, reaching a maximum of 0.07 g L^−1^ in only 2 days as opposed to 5 days and 0.06 g L^−1^ in the culture cultivated at 30 °C (Figure 2D).

**Figure 3:**
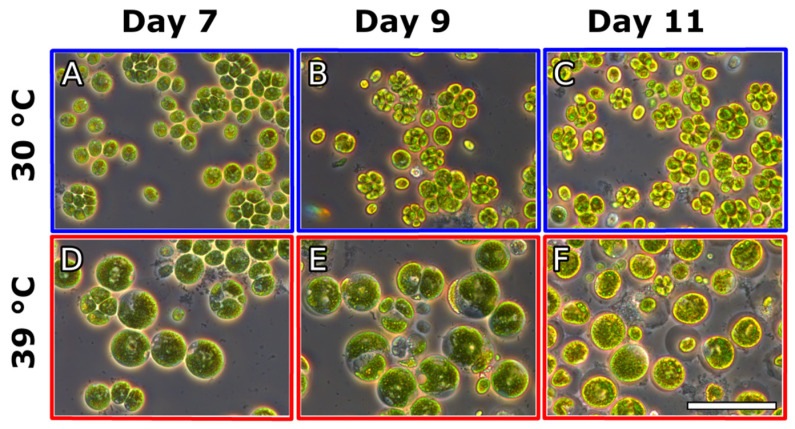
Effect of supraoptimal temperature on the cell division pattern and cell size in *C. reinhardtii*. The algal culture was cultivated for 6 days at 30 °C after which it was split, transferred to 30 °C (**A**–**C**) and 39 °C (**D**–**F**) and monitored for another 5 days. Day 7 corresponds to the first day after the split. Size of bar: 50 µm.

Microscopic observations of the culture cultivated at 30 °C did not reveal any change in the pattern of cell division with cells having a median cell volume of 422 µm^3^ and cell division occurring, as expected, during the course of the experiment with mother cells dividing predominantly into eight daughter cells (Figure 2B and Figure 3). In contrast, the cells transferred to 39 °C largely stopped dividing and the few dividing cells formed mostly two or four daughter cells. The inhibition of cell division was also reflected in the median cell volume, which increased eight-fold at 39 °C reaching 3479 µm^3^.

### 3.2. The Effects of Biomass Density

**Figure 4:**
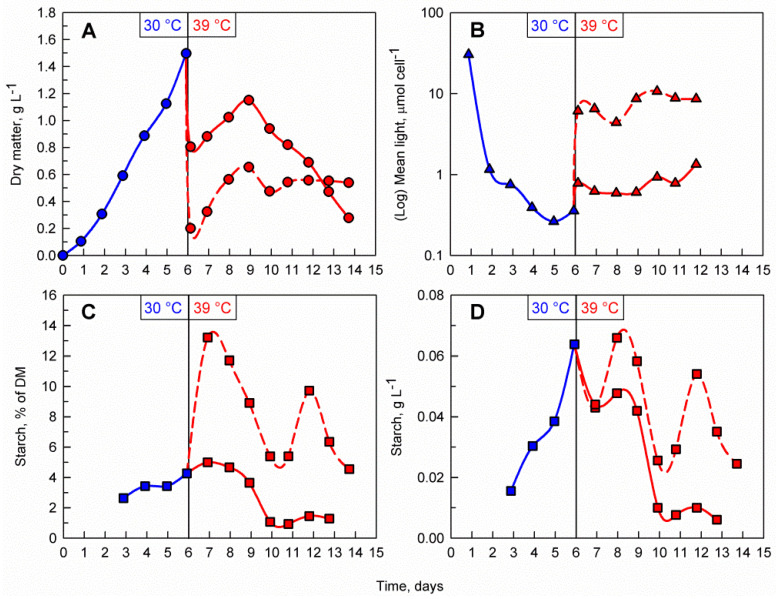
Effect of different biomass concentrations at the supraoptimal temperature on the time course of dry matter accumulation (**A**), mean light availability (**B**), biomass starch content (**C**), and volumetric starch concentration (**D**) in the culture. The vertical line at day 6 represents the shift between biomass accumulation and supraoptimal temperature phases. Blue lines and markers indicate cultivation at 30 °C and red lines and markers indicate cultivation at 39 °C. During the biomass accumulation phase a single *C. reinhardtii* culture was cultivated at 30 °C. After 6 days, the culture was split into two and diluted to 0.2 g L^−1^ (dashed red line) and 0.8 g L^−1^ (solid red line) then transferred to 39 °C.

Light availability within the culture suspension itself is a function of biomass density and incident light intensity. To study the effect of light availability on starch accumulation at a supraoptimal temperature, two parallel cultures of different biomass concentrations and identical incident light intensity were compared. To do so, a *C. reinhardtii* culture was cultivated at 30 °C for 6 days. After reaching a biomass concentration of 1.5 g L^−1^ the culture was diluted with a HS medium to avoid nutrient limitation and split into two cultures, with an initial biomass concentration of 0.2 g L^−1^ (less dense culture) and 0.8 g L^−1^ (dense culture); these were then cultivated at 39 °C for 8 more days (Figure 4A). During the first three days after dilution and transfer to 39 °C, both cultures increased in biomass and reached a maximum of 0.6 g L^−1^ and 1.1 g L^−1^, respectively. After this, the biomass concentration in the less dense culture remained constant while the biomass in the dense culture started to decline rapidly. The difference in biomass concentrations was also reflected in the mean light availability in the cultures, with the cells in the less dense culture being exposed to notably more light than the ones in the dense culture (Figure 4B). The increase in biomass relative starch content was remarkably rapid and was much more pronounced in the less dense culture, reaching a maximum of 13.2 % of DM within the first day of the transfer to 39 °C (Figure 4C). This rapid 3-fold increase from the initial culture represents a striking contrast to the starch values within the dense culture, which did not increase when compared to the pre-treatment phase. When comparing the different biomass concentrations in the cultures, the volumetric starch concentration (g L^−1^) in the less dense culture was about 20% higher than in the dense culture. However, the maximum volumetric starch concentration reached at 39 °C was similar (in the case of the less dense culture) or lower (in the case of the dense culture) than that reached during the biomass accumulation phase at 30 °C (Figure 4D). Similar experiments with similar results were also performed at two lower initial cell densities in 39 °C, 0.1 g L^−1^ and 0.2 g L^−1^ (Figure S1).

### 3.3. Transfer Back to Optimal Temperature

**Figure 5:**
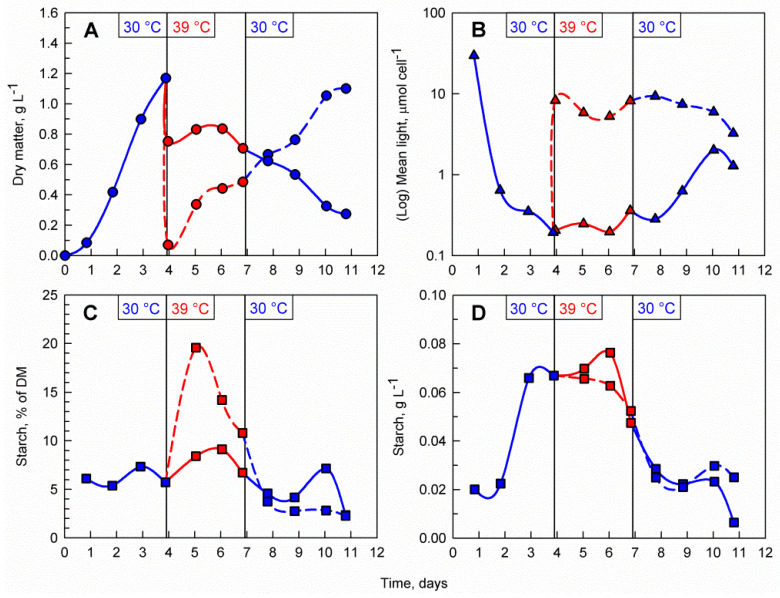
Effect of the combination of temperature shifts and different biomass concentrations on the course of dry matter accumulation (**A**), mean light availability (**B**), biomass starch content (**C**), volumetric starch concentration in the culture (**D**). A single *C. reinhardtii* culture was cultivated at 30 °C. After 4 days the culture was split into two, transferred to 39 °C and diluted to 0.1 mg mL^−1^ (dashed red lines) and 0.8 mg mL^−1^ (solid red lines), respectively. At day 7, the 0.1 mg mL^−1^ culture (dashed blue lines) and the 0.8 mg mL^−1^ culture (solid blue line) were transferred to 30 °C. Vertical lines at days 4 and 7 indicate those shifts in temperature (from 30 °C to 39 °C on day 4 and from 39 °C to 30 °C on day 7).

As previously demonstrated, the supraoptimal temperature treatment allows rapid accumulation of starch, during which time, the maximum biomass starch content is reached rapidly within 1 to 2 days. However, due to the nature of the temperature block on cell division, a biomass accumulation phase is required before the treatment can be applied. Thus, a possible starch production process on an industrial scale will involve a repeated-batch culture which is treated with consecutive alterations of temperature between 30 and 39 °C. To investigate whether the cells are viable, and their cell cycle block can recover after the supraoptimal temperature phase, the *C. reinhardtii* culture was cultivated at 30 °C for 4 days. After reaching a biomass concentration of 1.2 g L^−1^, the culture was diluted with a HS medium and split into two cultures with initial biomass concentrations of 0.1 g L^−1^ and 0.8 g L^−1^, respectively (Figure 5A). Immediately after dilution, both cultures were transferred to 39 °C for a period of three days. During the transfer to supraoptimal temperature, biomass accumulation in both cultures decreased with the decrease being much more pronounced in the 0.8 g L^−1^ culture. When the culture was moved back to 30 °C, biomass accumulation resumed in the 0.1 g L^−1^ culture while in the 0.8 g L^−1^ culture, biomass concentrations declined gradually. The difference in initial biomass concentration after dilution was also reflected in the mean light availability in the cultures, with the cells in the less dense culture being exposed to notably more light than the ones in the dense culture (Figure 5B). Starch synthesis was much more pronounced in the 0.1 g L^−1^ culture with biomass starch content increasing 4-fold and reaching nearly 20% of DM within the first day of transfer to supraoptimal temperature (compared to only 8% of DM in the 0.8 g L^−1^ culture) (Figure 5C). Although the biomass starch content in terms of percent of DM was higher in the 0.1 g L^−1^ culture, the volumetric starch concentration (g L^−1^) in both cultures was nearly identical due to the difference in biomass concentrations within the two cultures (Figure 5D).

**Figure 6:**
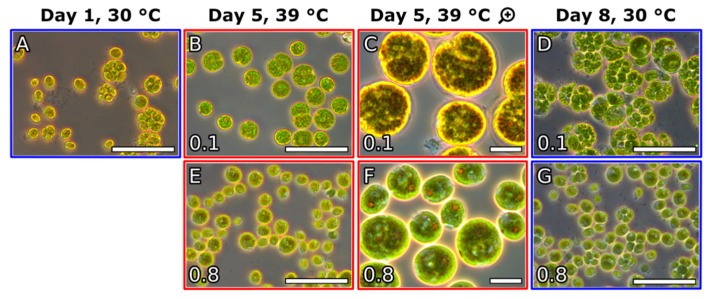
Microscopic observations. (**A**): Cells during day one of the biomass accumulation phase at 30 °C. (**B**,**E**): Cells one day after transfer to 39 °C and dilution to 0.1 g L^−1^ and 0.8 g L^−1^, respectively. (**C**,**F**): magnification and iodine staining of starch granules in the cells from panels (**B**,**E**), respectively. (**D**,**G**): cells one day after transfer back to 30 °C of the cultures originally diluted to 0.1 g L^−1^ and 0.8 g L^−1^. Size of bar on panels (**A**,**B**,**D**,**E**,**G**): 50 µm, panels (**C**,**F**): 10 µm.

Microscopic observations and analysis of the cell cycle confirmed that *C. reinhardtii* cells were dividing during the biomass accumulation phase at 30 °C (Figure 6A) and transfer to supraoptimal temperature resulted in a block of cell division (Figure 6B,C,E,F). The cells in the 0.1 g L^−1^ culture were larger and rounder as opposed to cells in the 0.8 g L^−1^ culture, which were smaller (Figure 6B,E). Staining with iodine revealed the presence of large amounts of starch granules in the chloroplasts of the cells in both cultures one day after the transfer (Figure 6C,F). Upon transfer back to 30 °C cell cycle progression was restored in the 0.1 g L^−1^ culture within one day (Figure 6D) as opposed to the cells in the 0.8 g L^−1^ which did not recover (Figure 6G).

## 4. Discussion

### 4.1. Effects of Supraoptimal Temperature on Starch Accumulation in C. reinhardtii at Pilot-Scale

The transfer of *C. reinhardtii* cultures from optimal to supraoptimal temperature proved to have a pronounced effect on both cell cycle progression and the accumulation of energy reserves in the form of starch. Upon 39 °C treatment, cell division was inhibited, mean cell size was increased, and the biomass starch content was enhanced more than 2-fold (Figure 2C, Figure 4C, Figure 5C, Figure S1C and Table 2). These observations are in agreement with results from supraoptimal temperature experiments conducted with synchronized cultures of *C. reinhardtii* in laboratory-scale experiments [6] and resemble the effect of supraoptimal temperature on *Chlorella* sp. [7,8]. The increase in starch as an energy storage molecule during a period of inhibited cell division supports the inverse relationship between chemical energy storage and energy expenditure for the normal operation of the cell cycle under optimal growth conditions. Increased starch accumulation in green algae is often linked to a block in the cell cycle. It was observed not only as an effect of supraoptimal temperature treatment but also as a response to nutrient deprivation [13,20,22,25,26] and for cell cycle gene mutants [10]. The starch (over)accumulation is genetically linked to mutations in *phosphoglucomutase* 1, an enzyme involved in both glycolysis and starch biosynthesis [19]. Furthermore, *C. reinhardtii* mutants in DYRK kinase were shown to hyper-accumulate both starch and oil [21].

Starch synthesis in response to nitrogen depletion in laboratory-scale experiments with *C. reinhardtii* cultures has been reported to increase starch content to up to 70 μg 10^−6^ cells (i.e., 70 pg cell^−1^). However, this type of treatment required 7 days of nitrogen starvation until the maximum starch content was reached [26]. Similarly, starch levels induced by sulfur depletion reached up to 49% of DM after 20 days. However, during the upscaling of the process, the maximum starch content which was reached was nearly two-fold lower at around 25% of DM [22]. Thus, the results produced under stable laboratory conditions are often difficult to directly extrapolate to an industrial scale. This is also well documented on the comparison of starch production in supraoptimal temperature in laboratory conditions [6] and the results described here, i.e., 80 pg cell^−1^ versus 16 pg cell^−1^. Notwithstanding, the limitation of pilot-scale cultivation, the starch accumulation in experiments presented here was significantly faster (only 1 to 2 days) compared to the nutrient limitation conditions [22,26]. Such a significant decrease in cultivation time might prove of notable importance for the economic viability of industrial-scale production of microalgal based starch.

### 4.2. The Importance of Light Availability

A modern biotechnological process requires high productivity and cost efficiency [27]. This can only be achieved through rapid accumulation and high volumetric concentrations of the desired product. In order for microalgal starch production to be economically viable, starch yields per volume of culture must be high. This is only possible through increased starch content per cell at high biomass concentrations. Yet, increase in biomass density led to a decrease in mean light availability within the culture (Figure 4B, Figure 5B and Figure S1B). This is likely due to light scattering and self-shading of the microalgal cells which have been found to cause sharp reductions in light availability with depth [28,29,30]. This limited light availability within the culture led to a reduction in the starch content and ultimately limited the effectiveness of the supraoptimal temperature treatment (Table 2). Similarly, recent findings in *Nannochloropsis* sp. showed a strong link between photosynthetic efficiency and the accumulation rate of lipids which are the primary energy storage compound in this microalga [31]. As a result, although the supraoptimal treatment led to a more rapid accumulation of starch, the maximum volumetric starch concentration achieved during cultivation at 39 °C did not show any notable increase over the maximum volumetric starch concentrations reached at 30 °C.

Furthermore, in the cultivation system used in this study, the combination of high cell density (leading to low light availability) and supraoptimal temperature seemed to have a negative effect on *C. reinhardtii* cultures after a certain period of time. In both cultivations at a starting biomass density of 0.8 mg mL^−1^, biomass decline was observed after 3 days of exposure to supraoptimal temperature (Figure 4A and Figure 5A), and the cultures did not recover when transferred to the optimal growth temperature (Figure 5A). In addition, light penetration into the high biomass cultures was further hindered by excessive biofilm formation (Figure S2).

When synthesis of a substance such as starch is linked to the photosynthetic capacity of the cell, and consequently to light availability, production in large-scale requires efficient utilization of light. In this regard, an effective reactor design that ensures proper light distribution within a high biomass culture is essential [28,29,32]. This can be achieved by decreasing the optical path that light has to travel within the culture and carefully controlling the biomass concentration at the time when supraoptimal temperature is applied.

### 4.3. Perspectives

The main advantage of the experiments presented here, compared to the other treatments, is the speed of starch accumulation (Table 2). In the context of large-scale cultivation of microalgae, short turnaround times are important not only because they allow for higher productivity but also because they reduce the risk of biological contamination by a competitive microalgal species or fortuitous grazers. This makes the supraoptimal temperature method a viable option for increasing starch productivity in microalgae.

Based on the results described here, a tentative industrial-scale starch production strategy based on the use of supraoptimal temperature can employ both batch and repeated batch modes of operation. This is made possible by the fact that the culture can be recovered and reused as an inoculum after the temperature treatment (Figure 5A and Figure 6). The production flow can consist of alternating biomass accumulation (6–7 days) and temperature treatment phases (1–2 days). Moreover, large and heavy cells that are filled with starch should enable better downstream processing of the biomass. However, the economic viability of the process depends greatly on improving bioreactor design and reducing the associated energy and labor costs.

## 5. Conclusions

The method of supraoptimal temperature treatment was successfully applied in pilot-scale and resulted in a considerable, nearly 3-fold, enhancement of starch content in *C. reinhardtii* at low biomass densities. Moreover, starch synthesis was faster, with the maximum being reached within only 1–2 days, compared to five days at the optimal temperature. Thus, the supraoptimal temperature treatment provides a viable alternative to other starch stimulating methods, such as nutrient depletion. This is especially true when times required for starch accumulation are taken into account. However, technical challenges, such as bioreactor design and improved light availability per cell, still need to be dealt with.

## Figures and Tables

**Table 1:** Overview of the experiments performed including date and duration. Temperature and initial biomass concentration after dilution of the culture from the biomass accumulation phase are shown. All experiments were performed in the summer of 2018.

Experiment Date	Total Duration (days)	Temperature Treatment (°C)	DM at the Beginning of Experiment (g L^−1^)
17.07–31.07	14	39	0.2
		39	0.8
13.08–23.08	10	39	0.1
		39	0.2
23.08–03.09	11	39	0.1
		39	0.8
03.09–17.09	12	30	0.1
		39	0.1

**Table 2:** Comparison of the effect of supraoptimal temperature in combination with various initial biomass concentrations on the maximum biomass starch content and the time when the maximum volumetric starch concentration was attained.

Initial DM (g L^−1^)	Temperature (°C)	Max. Biomass Starch Content (% of DM)	Max. Volumetric Starch Concentration (g L^−1^)	Time Required to Achieve Max. Volumetric Starch Concentration (Days)
0.1	39	21 *	0.067 *	1.3 *
0.2	39	14 **	0.069 **	2 **
0.8	39	7 **	0.060 **	2 **
0.1	30	8	0.057	5

* Shown value is an average of three cultivations; ** Shown value is an average of two cultivations.

## Data Availability

All data presented in this study are available within this article or Supplementary Materials. There are no special databases associated with this manuscript.

## Supplementary Materials

The following are available online at https://www.mdpi.com/article/10.3390/cells10051084/s1, Figure S1: Effect of the combination of supraoptimal temperature and different biomass concentrations (A) on % of starch in the microalgal biomass (B), mean light availability (C) and starch concentration in the culture (D). Figure S2: Cultivation vessels where *C. reinhardtii* cultures treated at a supraoptimal temperature and with different starting biomass densities have been cultured.
