# Supplementary material for: Starch Production in Chlamydomonas reinhardtii through Supraoptimal Temperature in a Pilot-Scale Photobioreactor
Source: Cells. 2021 May 1;10(5):1084. doi: 10.3390/cells10051084 (PMC8147326; doi:10.3390/cells10051084)
Supplement: Supplementary file 1 [file cells-10-01084-s001.zip › cells-1193382-supplementary.pdf]

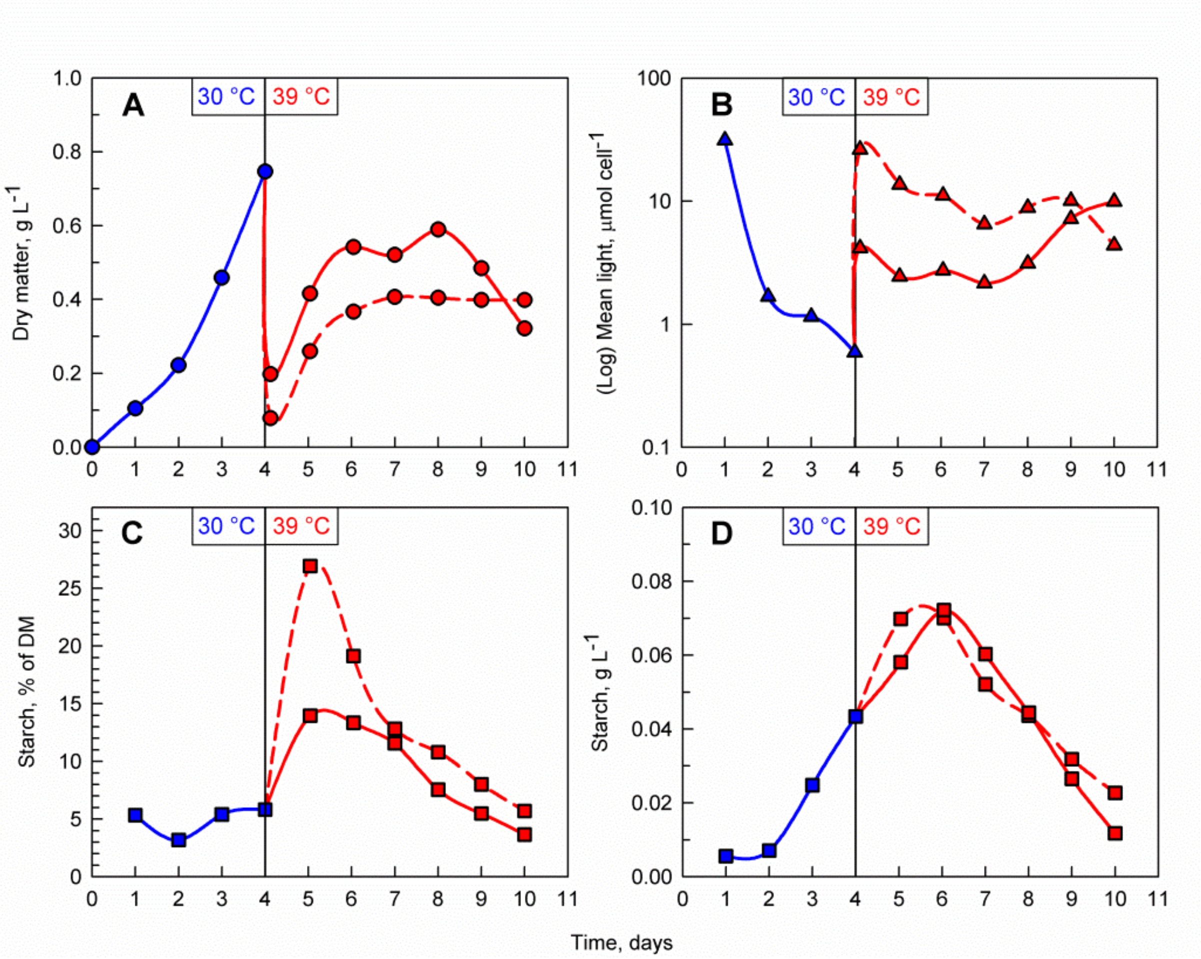

Figure S1. Effect of the combination of supraoptimal temperature and different biomass concentrations (**A**) on mean light availability (**B**), biomass starch content (**C**) and volumetric starch concentration (**D**) in the culture. The vertical line at day 4 represents the shift between biomass accumulation and supraoptimal temperature phases. Blue lines and markers indicate cultivation at 30 °C and red lines and markers indicate cultivation at 39 °C. During the biomass accumulation phase a single *C. reinhardtii* culture was cultivated at 30 °C. After 4 days the culture was split into two, transferred to 39 °C and was diluted to 0.1 g L^{-1} (dashed red line) and 0.2 g L^{-1} (solid red line) respectively.

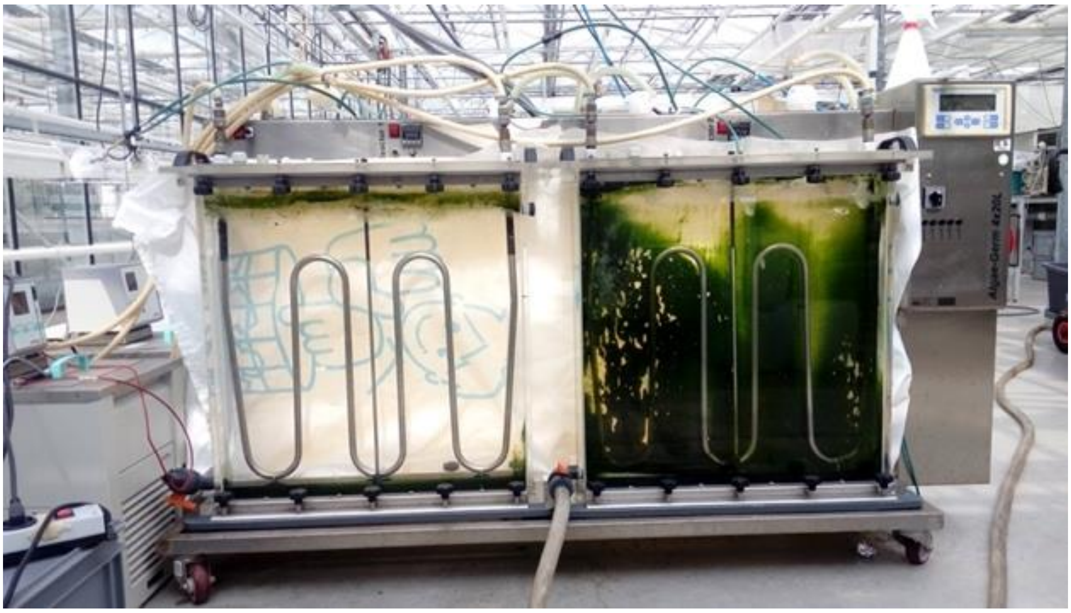

Figure S2. Cultivation vessels where *C. reinhardtii* cultures were incubated at the supraoptimal temperature and with various initial biomass concentrations. The photograph was taken moments after the culture suspension from both panels was discarded. Left panel: culture with biomass density of 0.1 g L^{-1}. Right panel: excessive bio film formation on the back wall where culture with biomass density of 0.8 g L^{-1} was cultivated.
